# Dignity promotion in people with advanced chronic diseases: contributions for a value-based healthcare practice

**DOI:** 10.3389/fpubh.2023.1156830

**Published:** 2023-07-27

**Authors:** Carlos Laranjeira

**Affiliations:** ^1^Department of Nursing Science, School of Health Sciences of Polytechnic of Leiria, Leiria, Portugal; ^2^Centre for Innovative Care and Health Technology (ciTechCare), Polytechnic of Leiria, Leiria, Portugal; ^3^Comprehensive Health Research Centre (CHRC), University of Évora, Évora, Portugal

**Keywords:** dignity, chronic disease, identity, virtue, value-based healthcare, ethics

## 1. Introduction

Since human dignity interpretations depend on culture and fluctuate over time, they are historically, politically, and culturally rooted. The term dignity comes from the Latin terms *dignitus*, which means competent, and *dignus*, which means derived worth (1). Even though the phrase “human dignity” has been used in several research, its definition remains unclear (2). A basic definition of human dignity is an individual's intrinsic value as a result of being human. The United Nations [(3), p. 1948] emphasizes that all human beings have inherent dignity: “All human beings are born free and equal in dignity and rights.” Individuals develop a feeling of this value via their interactions with others (4). Human dignity has long been valued in all countries and faiths and serves as the cornerstone for human rights (5). Professional codes of conduct for healthcare workers promote care that safeguards patients' dignity and emphasizes that respect for others is a fundamental human right. At this point, the International Council of Nurses' (ICN) Code of Ethics [(6), p. 1] states “inherent in nursing is a respect for human rights, including cultural rights, the right to life and choice, to dignity and to be treated with respect.”

According to some researchers, it is often simpler for practitioners to express undignified care than it is to define dignified care (7, 8). Illness trajectory, power imbalance, a loss of privacy, and being treated or hospitalized can all have an impact on human dignity. Human dignity violations can harm patients' bodies, spirits, morality, and spirituality, exposing them to stress and anguish (9). As we become older, we want to know that we will be loved and respected, that the person taking care of us is compassionate and understands that we are unique individuals with important lives. We seek attention. A growing number of patients are given life-limiting illnesses each year. Responsive care is ethically necessary since people with advanced diseases depend on others for their wellbeing. We determined that the foundation of what patients and their caregivers believe to be responsive care is the preservation and advancement of what Nordenfeldt (10) refers to as the “dignity of identity.”

Human dignity is linked to the property of being human, and it is critical in health, medicine or healthcare systems (11). Chronic patients may be among the most vulnerable social groups, since they have not only lost physical capacities, but also face unique psychological, social, and economic demands as a result of their condition. Healthcare practitioners must maintain open lines of contact with patients while also respecting their personal rights and professional principles such as human dignity, as well as being sensitive to existing disparities (12). Respect for patients' dignity is essential in their care and improves their quality of life. Furthermore, healthcare system-related characteristics such as patient-provider communication may promote drug adherence, as well as patient dignity (13). As a result, the researchers looked at the possible link between human dignity preservation and incentives to promote patient adherence to medical advice.

This essay discusses the value of dignity and personhood for those suffering from life-limiting illnesses such as advanced chronic diseases. Before discussing our theoretical framework for comprehending the contextual character of the dignity of identity, we first give background literature on the relational operations of dignity of identity. Increasing professionals' ethical awareness allows them to see the complexities of patient requirements and circumstances, from which possible ethical conflicts eventually arise, and address them. This is one way to empower professionals to behave as moral agents and provide patients with safe and ethical care. Amidst public interest in the tragic outcomes of the COVID-19 pandemic, this paper is a timely reminder of the relevance of ethical awareness of healthcare professionals and the need to foster human dignity as the last goal pursued by value-based health care.

## 2. Dignity in a historical perspective

No other notion has had as much resonance in the history of ethical philosophy as the concept of human dignity. However, defining the term has resulted in intense scholarly arguments in a variety of fields of moral reflection (14). We all know that the concept of human dignity is quite old and has its origins in classical culture. Cicero's *dignitas romana* mirrors a notion already known to Greek philosophy, however, the focus in these texts is mostly on the socio-political dimensions of personal activity within the society. The essence of a person's dignity is the dignity with which he builds and lives his life in society. Against this historical backdrop, Christianity examines another aspect of human dignity in-depth, offering a more specific anthropological viewpoint. A theological definition of human dignity must include a reference to the notion of the *imago Dei*.

Despite philosophical and disciplinary differences, researchers conceptualize dignity as dualistic (15), so there are essentially two approaches to dignity (16). One approach is to think of it as intrinsic and ontological, or as what some writers refer to as fundamental or absolute dignity. According to this perspective, dignity is an unchangeable aspect of personality that does not change or rely on the situation. Absolute dignity refers to principles such as human worth, freedom, responsibility, and helping others that are impossible to compromise. The second viewpoint speaks of what is referred to as dynamic or relative dignity, which is a characteristic of a person that is connected to how they view themselves and the environment in which they live, is impacted by culture, and has both rigid and flexible values. On the other hand, relative dignity derives from cultural factors in which individuals find themselves, such as educational level and social network (17, 18). In the absence of these cultural conditions, there is a risk of violation of dignity, so health professionals must be aware of the ethical principles, values and actions necessary for the maintenance/promotion of dignity. What dignity means in practice will depend on how patients see themselves and are perceived by others, as well as how the nature of the illness in question affects the person's life and identity. Nonetheless, dignity is generally thought to be a fundamentally intrinsic feature of the human individual (19).

Dignity is also a cultural concept, whose definition and maintenance is both social and culturally dictated (20). Individual attitudes, values, and perceptions shape an individual's norms and expectations about the maintenance of dignity (21). However, the term “dignity” intersects with other terms such as pride, self-respect, quality of life, wellbeing, hope, self-esteem, autonomy, respect, empowerment, and communication.

To advance thinking on the matter, a more responsive concept of dignity is required, one capable of capturing individual perspectives of personhood over time and in diverse settings. In this sense, the Ring Theory of Personhood (RToP) (22, 23) is widely used to evaluate current ideas on dignity. The RToP is composed of four concentric and interrelated rings: —the Innate, Individual, Relational, and Societal rings. A core feature of the RtoP is the fluid nature of interactions between the rings, which capture the dynamic/evolving nature of self-concepts of personhood (24). In order to construct domain-based identities, which in turn shape personal concepts of dignity, each ring incorporates particular beliefs, moral values, ethical principles, family mores, cultural norms, attitudes, thoughts, decisional preferences, duties, and obligations. Thus, giving primacy to a patient's personhood and dignity requires that professionals attend very carefully to the meaning of the illness.

### 2.1. Dignity in patients with advanced chronic diseases

Chronic diseases such as heart disease, cancer, and diabetes are major and universal public health problems. Poor health status, failing bodies, an increase in symptom burden, functional incapacity, and cognitive decline are all consequences of declining physical function brought on by chronic diseases (25, 26). Patients with chronic diseases spend more of their lives with limitations induced by the disease, have higher symptom severity (27, 28), and must cope with unexpected mortality. Even though everyone will eventually die, losing one's dignity does not only happen at the end of life (25). Many of these patients feel agitated and have limited control over their symptoms, especially in the later stages of the disease. As a result, the major objective of care for individuals is to increase their life expectancy. As a result, palliative care is crucial for these patients, and one of the fundamental components of this palliative care is respect for dignity and human rights, regardless of nationality, ethnicity, religion, color, age, gender, disabilities, or socio-political circumstances. There is increasing research on dignity using several instruments, particularly the Patient Dignity Inventory, a rigorous tool that has been translated into many languages (29). Identifying and enhancing the patient's dignity can boost their confidence and contentment with treatment, enhance care, minimize hospitalization length, and improve patient outcomes. In contrast, the loss of a patient's dignity can have a negative impact on the patient's physical and mental health (30).

Dignity-conserving care offers an approach that clinicians can use to explicitly target the maintenance of dignity as a therapeutic objective and as a principle of bedside care for patients nearing death (31). In the challenge launched by this author, current Medicine must dedicate itself to a culture of caring (to advance a culture of caring). In clinical practice, dignity must be the value, not a value, capable of overestimating personhood (relating to the person, personality) over the so usual and undignified patient hood (relating to the strict condition of being sick, to the disease) (32). For this author, clinical practice should focus on the sick person and not on the person's diseases, following the care philosophy of Cicely Saunders of making the disease peripheral to the person. If the patient's dignity is not upheld, needless and maybe excruciating suffering will befall them (33). In this sense, the caregiver has an ethical obligation to ensure that the patient is treated as a friend and fellow human being when they come into contact. According to Lévinas responsibility ethics entails that one is not free in relation to the other and that the ethical need has an endless scope (33).

Harvey Chochinov, promoter of the concept of the tonality of care (care tenor), developed the mnemonic A, B, C, and D of Conservative Care of Dignity (34) in analogy to the A, B, C of cardio-respiratory resuscitation (A-airway, B- breathing, and C-circulation). In this work, the author postulates that all health professionals dedicated to the holistic care of the person and their dignity must develop specific skills that can be listed through the simple mnemonic A (Attitude); B (Behavior); C (Compassion), and D (Dialogue). This entails being sensitive to and respecting the presented concerns, aspirations, and goals (35, 36). Individualized care, restoration of control, respect, advocacy, and careful listening are all required for dignity in care. Other variables that support dignity include a caring culture, staff attitudes and behavior, and the performance of specialized care tasks (15). Along with more tangible and precise care acts, perceptiveness, openness, listening, and respect are elements of a complete approach to dignity-conserving care for patients (35). As a result, enhancing one's sense of self involves conserving it or regaining it, sustaining one's identity, and ensuring and maintaining one's self-esteem. Self-responsibility, participation in decision-making regarding their own treatments and care, attention to patient rights (37), and cultivating a pedagogical climate of mutual understanding all supported the autonomy of people with chronic progressive disease.

Health professionals also play a crucial role in lowering stigma views and helping people manage stigma, which helps them get support by protecting them from shame. Health professionals must reassure patients that their inner sense of self is unaffected by their illness and that they will always be the same person in order to provide them with a feeling of continuity in their identity. Improvements in patients' self-esteem, a deeper understanding of their purpose and meaning in life, maintaining and improving their quality of life, and providing relief from multifaceted distress through the interaction between patients and nurses are all necessary outcomes of dignity-centered care (25). Recognizing these dynamics helps understand how patients retain their dignity when facing changes and losses, and can help deliver proactive and dignity-sustaining care to lead patients through the illness's trajectory (38). Indeed, some factors should be addressed to uphold a person's dignity (Table 1).

**Table 1:** Core principles to support dignity in patient care.

Throughout every stage of human existence, value and accept each person as a unique individual “being seen and being heard” (39).
Safeguard and respect each individual's right to self-determination as a fundamental human right (39).
Protect and promote patient autonomy by respecting their needs, preferences, alternatives, beliefs/values, privacy, and advocating against stigma (25, 40).
Acknowledge and guarantee that the rights and best interests of the incapacitated are central to all decision-making processes pertaining to their care and welfare. Open communication, patient engagement in developing care plans, and shared decision-making can minimize many ethical conundrums, especially in end-of-life care (41).
In the quest for more dignified treatment, provide knowledgeable, empathetic, compassionate, and non-possessive care (40).
To avoid undignified treatment, promote advanced care planning by people with chronic progressive diseases (25).

The challenge is therefore to combine the importance of obtaining the best clinical results with the need to satisfy the specific expectations of each individual, simultaneously ensuring that the necessary resources are organized and used, and the costs involved are managed, as far as that is strictly necessary. The concept of value in health care allows for greater involvement and accountability of all stakeholders, from health systems and organizations to professionals and citizens themselves, who thus contribute, in synergy and actively, to improving the health of each person and optimize the functioning of health systems (42).

## 3. Final remarks

Although present in several codes of conduct and standards for patient care, dignity is still a contentious idea that is hard to define, quantify, and apply to the field of healthcare. Dignity is a value-based and humanistic concept that emerged within the field of ethics, associated with the main attributes of personhood (intrinsic), sociability (relational/behavioral), respect, and autonomy. RToP allows healthcare professionals to determine which of the Innate, Individual, Relational, and Societal rings dominate thinking and need greater attention at a particular moment and context. Developing ethical awareness can empower professionals to act as moral agents in providing patients with safe and ethical care. An ethical awareness knows no enemies, no strangers. It represents an integrating and regulating principle that can establish relationships between particular issues and the universalist and formal claim, without the intention of becoming, itself, absolute. In fact, it could not feed this absolutist claim since, as an integrating principle, its validity is restricted to the attempt to interpret transcendental moral expectations for the context of individuals, on whose agreement, ultimately, it will depend. This ethical consciousness is a dialectical process of the reflected balance between the places (physical environment and organizational culture), the people (the attitudes and behavior of professionals and others), and the processes (care activities) which are crucial to foster value-based healthcare practices.

## Author contributions

The author confirms being the sole contributor of this work and has approved it for publication.
